# Socio-demographic characteristics, lifestyle factors, multi-morbid conditions and depressive symptoms among Nepalese older adults

**DOI:** 10.1186/s12888-020-02680-3

**Published:** 2020-05-26

**Authors:** Uday Narayan Yadav, Tarka Bahadur Thapa, Sabuj Kanti Mistry, Roshan Pokhrel, Mark Fort Harris

**Affiliations:** 1 Centre for Primary Health Care and Equity, University of New South Wales, Sydney, Australia; 2 Forum for Health Research and Development, Dharan, Nepal; 3 James P Grant School of Public Health, BRAC University, 68 Shahid Tajuddin Ahmed Sharani, Mohakhali, Dhaka, 1212 Bangladesh; 4 Department of Health Services, Ministry of Health and Population, Kathmandu, Nepal

**Keywords:** Depressive symptoms, Chronic disease, Socio-demographic characteristics, Lifestyle factors, Older adults

## Abstract

**Background:**

Depressive symptoms (DS) are a well-recognized public health problem across the world. There is limited evidence with regard to DS and its associates, such as socio-demographic characteristics, lifestyle factors and chronic conditions in low-income countries like Nepal. In this study, we aimed to assess the level of DS and its relationship with socio-demographic characteristics, lifestyle factors and chronic disease conditions among community dwelling older people in Nepal.

**Methods:**

We conducted a cross-sectional study of 794 older adults aged 60 or above residing in the rural setting of the *Sunsari* and *Morang* districts of eastern Nepal between January and April 2018. Multi-stage cluster sampling was adopted to select the study participants. Data included socio-demographics, lifestyle factors, self-reported chronic disease conditions and the Geriatric depression scale. On Geriatric depression scale, an older adult with a test score greater than five were defined as having depressive symptoms. Determinants of DS were estimated through the generalized estimating equation (GEE) approach by considering exchangeable correlation structure among clusters.

**Results:**

In our study samples, nearly 55.8% of the older adults were found to be suffering from DS. We found a significant association between DS and being female (aOR: 1.25, 95% CI: 0.89–2.09), Buddhism (aOR: 1.95, 95% CI: 1.58–2.42), *Dalits* (aOR: 2.60, 95% CI: 1.19–5.65), unemployed, low family income (aOR: 1.77, 95% CI: 1.07–2.92), smokers (aOR: 1.49, 95% CI: 1.01–2.20) and having chronic multi-morbid conditions (aOR: 1.67, 95% CI: 1.09–2.55).

**Conclusions:**

The prevalence of DS was high among community-dwelling older adults in eastern Nepal. Our findings suggest the need for **mental health prevention and management programs targeting** the older population in rural Nepal.

## Background

Depressive symptoms (DS) are a well-recognized public health problem throughout the globe, and older adults are more likely to suffer from depressive symptoms (DS) [1]. The data from World Health Organization (WHO) have recognized DS as the most common mental disorder affecting approximately 7% of the population worldwide [2]. However, mental health among older adults has not been a priority area in many low-income countries [3]. Epidemiological studies from South Asian countries reported a high prevalence of depression among older people, ranging from 34.4% in India [4], 40.6% in Pakistan [5], and 44.2% in Bangladesh [6]. A recent study from Nepal showed, 60.6% of older adults had DS [7]. Despite its higher prevalence, there is poor access to early diagnosis and treatment of depression is generally held back because of social stigma with mental health issues and insufficient screening and treatment services in primary health care [8, 9]. Symptoms of depression are not usually recognized by families, caregivers or by older adults themselves because of a lack of understanding of the condition, shame or denial that these conditions need medical care and a perception that DS are incurable in Nepal.

Various short and long-term factors across the life cycle influence the development of DS. The presence of chronic disease and socio-demographic factors such as age, gender, illiteracy, income level, marital status, mistreatment (which implies deliberate neglect, abuse or harm) and lifestyle behaviors (e.g., physical inactivity, smoking or chewing tobacco products, use of excessive alcohol) play a role in the development of DS [5, 10–12]. Early screening and management of depression can improve the quality of life of older adults [1]. However, healthcare systems in low income countries like Nepal are not able to deal with mental health problems including DS among this target population. Nepal is a signatory to the Comprehensive Mental Health Action Plan (2013–2020) and to the Sustainable Development Goals (2015–2030), which aim to reduce mental health problems through implementing strategies for promotion and prevention, and providing comprehensive and social care in community-based settings [13, 14]. However, performance falls well short of these objectives.

To date, there have been no systematically conducted studies in rural parts of south-eastern Nepal where most of the marginalised communities (Madhesi, Dalits and Indigenous groups) resides. In this line, this study could provide evidence to guide policies and programs to tackle this overwhelming problem. Hence, the present study aimed to address a critical knowledge gap by assessing the level of DS and its relationship with socio-demographic characteristics, lifestyle factors and multi-morbid conditions among community dwelling older adults of *Morang* and *Sunsari* district (Province One), Nepal.

## Methods

### Study design and participants

A community-based cross-sectional study among older adults aged 60 or above was conducted between January and April 2018. A multi-stage cluster sampling approach was adopted to select study subjects in the rural setting of the *Morang* and *Sunsari* districts. Primary data was collected from 794 older Nepalese people adults’ aged ≥60 years. In the first stage, eight Rural Municipalities (RMs) were randomly selected from the list of RMs within each of the *Morang* and *Sunsari* districts (four from each district). Second, five wards in each of the selected RMs were randomly selected. Finally, samples were selected randomly from the list of eligible subjects and were interviewed by the trained interviewers. The detailed methodology of this study has been published previously [15].

### Measures

#### Socio-demographics and lifestyle factors

A semi-structured questionnaire was used to collect information on socio-demographic profiles (age, gender, religion, literacy status, marital status, family type) and behavior and lifestyle characteristics (history of smoking, tobacco, alcohol and physical activity) from the study participants. Socio-demographic and lifestyle variables are described in our previous work that is published by Yadav et.al [16].

#### Chronic disease history

Data was collected on the presence of Diabetes, Cardiovascular disease, Arthritis and Chronic Obstructive Pulmonary disease from respondents (at interview). Information on self-reported chronic disease was then verified either from medical records or by asking to see any prescribed medicines which patient was taking for the relevant condition.

#### Depressive symptoms (DS)

The 15-item Geriatric Depression Scale (GDS-15) was used to assess depression symptoms among our study population [17]. This instrument has previously been validated and used in Nepalese older population [18]. Scores range from 0 to 15. A test score from 0 to 5 is considered normal and patients with test scores greater than 5 were assessed as having depressive symptoms. Factor analysis was performed and the Cronbach’s alpha for GDS was found to be 0.81, indicating high consistency.

### Data collection

The study used both self-constructed semi-structured questionnaire and a structured validated short form of the Geriatric Depression Scale (GDS) to collect the information. The semi-structured tool was constructed through a rigorous literature review and with input from an expert already working in the field of geriatric health. The English version of the questionnaire was first translated into Nepali and back translated into English to check the consistency. Pretesting of the questionnaire was carried on a similar group of participants (*n* = 10) to assess its acceptability and validity before the study began. The data was collected by four trained enumerators (having clinical skills with bachelor’s degrees in public health) via face to face interviews with the subjects in the community setting. The study received ethics approval from Nepal Health Research Council, Nepal. A written informed consent form was obtained from each study participants prior to interview.

### Statistical analysis

Descriptive analysis was performed to assess the distribution of the variables. The χ2 test was employed to compare the prevalence of DS within different categories of a variable with 5% level of significance. Since the survey data were nested in nature with variations among clusters (municipalities), we used a mixed-effect logistic regression model to determine the true association between DS and associated factors. We considered socio-demographic, lifestyle and chronic disease variables as fixed effects and cluster variation as random effects. The parameters of the model were estimated through the generalized estimating equation (GEE) approach by considering exchangeable correlation structure among clusters [19]. The full model was run with those variables showing *P* < 0.25 in the unadjusted analysis. We used 2 models for multivariable modeling: in model 1, we analyzed the association between DS and socio-economic as well as lifestyle variables, whereas in model 2 we analyzed the association between DS and chronic diseases adjusted for both socio-demographic and lifestyle factors. Both unadjusted and adjusted odds ratios (ORs) were reported with 95% confidence intervals (95% CI). All analyses were performed using the statistical software Stata (Version 13.0).

## Results

### Background characteristics

A total of 794 people aged 60 years or above participated in the study with a mean age of 69.9 years, where 55.4% of the participants were aged between 60 and 69 years and 15% aged 80 years or above. As summarized in Table 1, the male-female ratio was similar among the study population (50.4 and 49.6%, respectively). A majority of the participants were Hindu (78.7%) and illiterate (80.1%). Nearly 38% of the participants were of Indigenous origin, and nearly 20% were *Dalit*. Only around half of the older adults (53.5%) reported being married at the time of the survey. In terms of occupational status, 54.2% of the people reported not being involved in any type of income generating activities. As such, around half of the participants had a family income of 5000 NRs or less. Of the participant’s, 62.2% reported smoking history and 48.2% used to consume smokeless tobacco. In addition, 36.5% of the participants reported alcohol consumption, and 77.1% did not take part in any physical exercise (Table 1). Moreover, the prevalence of Osteoarthritis, CVD, Diabetes and COPD was 41.7, 2.39, 5.3, 14.6% respectively and 14.6% of the participants were suffering from at least two of these comorbidities (Table 2).

**Table 1:** Socio-demographic characteristics and status of depressive symptoms among participants

	Total (*n* = 794)	%	Depressive symptoms				
			No (*n* = 351)	%	Yes (*n* = 443)	%	*p*-value
Age group (in years)							
60–69	440	(55.42)	201	(45.68)	239	(54.32)	0.224
70–79	235	(29.60)	106	(45.11)	129	(54.89)	
≥ 80	119	(14.99)	44	(36.97)	75	(63.03)	
Gender							
Male	400	(50.38)	201	(50.25)	199	(49.75)	0.001
Female	394	(49.62)	150	(38.07)	244	(61.93)	
District							
*Morang*	404	(50.88)	202	(50.00)	202	(50.00)	0.001
*Sunsari*	390	(49.12)	149	(38.21)	241	(61.79)	
Religion							
Hinduism	625	(78.72)	281	(44.96)	344	(55.04)	0.356
Buddhism	19	(2.39)	7	(36.84)	12	(63.16)	
Islam	125	(15.74)	49	(39.20)	76	(60.80)	
Christianity	25	(3.15)	14	(56.00)	11	(44.00)	
Ethnicity							
*Brahmin/Chettri/ Thakur*	69	(8.69)	50	(72.46)	19	(27.54)	
Indigenous	298	(37.53)	138	(46.31)	160	(53.69)	< 0.001
*Dalit*	157	(19.77)	53	(33.76)	104	(66.24)	
*Madhesi and other ethnic groups*	270	(34.01)	110	(40.74)	160	(59.26)	
Marital status							
Married	425	(53.53)	207	(48.71)	218	(51.29)	0.006
^1^Others	369	(46.47)	144	(39.02)	225	(60.98)	
Literacy							
Illiterate	636	(80.10)	264	(41.51)	372	(58.49)	0.002
literate	158	(19.90)	87	(55.06)	71	(44.94)	
Past occupation							
Employed	364	(45.84)	198	(54.40)	166	(45.60)	< 0.001
Unemployed	430	(54.16)	153	(35.58)	277	(64.42)	
Family monthly income							
Nrs. <= 5000	381	(47.98)	168	(44.09)	213	(55.91)	0.039
Nrs. 5000 < = 10,000	145	(18.26)	52	(35.86)	93	(64.14)	
Nrs. > 10,000	268	(33.75)	131	(48.88)	137	(51.12)	

^1^Others denotes widow/widower/divorced/separated/unmarried. ^2^Suffering from at least two chronic illnesses of osteoarthritis, CVD, diabetes and COPD

**Table 2:** Lifestyle characteristics, chronic conditions and status of depressive symptoms among participants

	Total (*n* = 794)	%	Depressive symptoms				
			No (*n* = 351)	%	Yes (*n* = 443)	%	*p*-value
Smoking habit							
Never smoker	300	(37.78)	151	(50.33)	149	(49.67)	0.007
Having smoking history	494	(62.22)	200	(40.49)	294	(59.51)	
Tobacco chewing habit							
Never tobacco chewer	411	(51.67)	186	(45.26)	225	(54.74)	0.538
Having tobacco chewing history	383	(48.24)	165	(43.08)	218	(56.92)	
Alcohol drinking habit							
Never drinker	504	(63.48)	220	(43.65)	284	(56.35)	0.678
Having alcohol drinking history	290	(36.52)	131	(45.17)	159	(54.83)	
Physical activity							
No physical exercise at all	612	(77.08)	255	(41.67)	357	(58.33)	0.008
Daily physical exercise	182	(22.92)	96	(52.75)	86	(47.25)	
Osteoarthritis							
No	463	(58.31)	241	(52.05)	222	(47.95)	< 0.001
Yes	331	(41.69)	110	(33.23)	221	(66.77)	
CVD							
No	775	(97.61)	348	(44.90)	427	(55.10)	0.012
Yes	19	(2.39)	3	(15.79)	16	(84.21)	
Diabetes							
No	752	(94.71)	333	(44.28)	419	(55.72)	0.856
Yes	42	(5.29)	18	(42.86)	24	(57.14)	
COPD							
No	678	(85.39)	318	(46.90)	360	(53.10)	< 0.001
Yes	116	(14.61)	33	(28.45)	83	(71.55)	
^2^Multiple morbidities							
No	678	(85.39)	318	(46.90)	360	(53.10)	< 0.001
Yes	116	(14.61)	33	(28.45)	83	(71.55)	

^1^Others denotes widow/widower/divorced/separated/unmarried. ^2^Suffering from at least two chronic illnesses of osteoarthritis, CVD, diabetes and COPD

### Prevalence of depressive symptoms

Overall, 55.8% of the participants were suffering from DS. Univariate analysis revealed that, DS were significantly higher among female, participants from *Sunsari* district, Buddhist, *Dalit Madhesi* ethnicity, illiterate, unemployed and participants with low family income (Table 1). Prevalence was also higher among those who smoke, do not exercise, and suffer from Osteoarthritis, CVD, COPD, multiple morbidities (Table 2).

### Association of risk factors with depressive symptoms

In the unadjusted analyses, age, gender, religion, ethnicity, marital status, literacy, occupation, family income, smoking history, physical exercise and having multiple morbidities were shown as moderately to highly significant in association with DS (Table 3). However, after adjusting all the potential covariates in the multiple logistic regression model, only gender, religion, ethnicity, occupation, family income, smoking history and having multiple morbidities remained as significant risk factors for DS at 5% level of significance.

**Table 3:** Multiple logistic regression model of the determinants of DS

	Crude			Adjusted^a^		
	OR	*p*-value	95% CI	OR	*p*-value	95% CI
Age group (in years)						
60–69	1.00			1.00		
70–79	1.09	0.709	0.69–1.70	0.91	0.704	0.55–1.50
> = 80	1.76	**0.028**	1.06–2.93	1.38	0.229	0.82–2.33
Gender						
Male	1.00					
Female	1.70	**0.000**	1.37–2.11	1.61	**0.000**	1.25–2.09
District						
*Morang*	1.00			1.00		
*Sunsari*	1.59	0.220	0.80–3.31	1.07	0.891	0.43–2.67
Religion						
Hinduism	1.00			1.00		
Buddhism	1.78	**0.000**	1.60–1.98	1.95	**0.000**	1.58–2.42
Islam	1.13	0.499	0.79–1.62	1.01	0.968	0.55–1.87
Christianity	0.92	0.623	0.67–1.27	1.22	0.198	0.90–1.65
Ethnicity						
*Brahmin/Chettri/Thakur*	1.00			1.00		
*Aadiwasi/Janjatis*	1.85	0.058	0.98–3.49	1.80	0.131	0.84–3.85
*Dalit*	2.80	**0.002**	1.47–5.35	2.60	**0.016**	1.19–5.65
*Madheshi and other ethnic groups*	2.04	**0.005**	1.23–3.37	1.82	0.127	0.84–3.93
Marital status						
Married	1.00			1.00		
^1^Others	1.63	**0.001**	1.24–2.15	1.16	0.151	0.95–1.41
Literacy						
Literate	1.00			1.00		
Illiterate	1.67	**0.041**	1.02–2.72	1.38	0.176	0.86–2.21
Occupation						
Employed	1.00			1.00		
Unemployed	2.28	**0.000**	1.76–2.97	2.05	**0.000**	1.47–2.87
^2^Income						
Nrs. > 10,000	1.00			1.00		
Nrs. 5000 < = 10,000	1.71	**0.014**	1.11–2.62	1.77	**0.025**	1.07–2.92
Nrs. <= 5000	1.43	**0.036**	1.02–2.01	1.41	0.077	0.96–2.08
Smoking habit						
Never tobacco user	1.00			1.00		
Having tobacco use history	1.39	**0.040**	1.02–1.92	1.49	**0.047**	1.01–2.20
Tobacco chewing habit						
Never tobacco chewer	1.00			Not taken in the model		
Having tobacco chewing history	0.94	0.640	0.74–1.20			
Alcohol drinking habit						
Never drinker	1.00			Not taken in the model		
Having alcohol drinking history	0.94	0.596	0.77–1.17			
Physical activity						
No physical exercise at all	1.00			1.00		
Daily physical exercise	1.48	0.234	0.78–2.82	0.96	0.894	0.53–1.73
Multi-morbidity						
No	1.00			1.00		
Yes	1.73	**0.009**	1.14–2.61	1.67	**0.018**	1.09–2.55

Significant *p*-values are bolded. ^2^Others denotes widow/widower/divorced/separated/unmarried. ^2^100 Nrs approximates 1 US Dollar. *Abbreviation*: *CVD* Cardiovascular disease, *COPD* Chronic Obstructive Pulmonary Disease

It was found that, female participants had 61% higher odds of suffering from DS compared to their male counterparts (aOR: 1.25, 95% CI: 0.89–2.09). An individual of the Buddhist community had around 1.9 times higher odds of suffering from DS compared to a Hindu person (aOR: 1.95, 95% CI: 1.58–2.42), and compared to people of *Brahmin/Chettri/Thakur* origin, an individual from *Dalit* community had 2.6 times higher odds of suffering from DS (aOR: 2.60, 95% CI: 1.19–5.65). Notably, an unemployed person had nearly two times higher odds of suffering from DS compared to a person engaged with any income generating activities (aOR: 2.05, 95% CI: 1.47–2.87). Participants with a smoking history had around 50% higher odds of suffering from DS than those who never consumed tobacco (aOR: 1.49, 95% CI: 1.01–2.20). Economic status was also identified as a prime risk factor as the participants with a family income of 5000–10,000 NRs had 77% higher odds of suffering from DS than those with a family income of 10,000 NRs or high (aOR: 1.77, 95% CI: 1.07–2.92). Also, a person suffering from multiple morbidities had 67% higher odds of suffering from DS than those with no multiple morbidities (aOR: 1.67, 95% CI: 1.09–2.55).

## Discussion

This study was an investigation that demonstrates an association between socio-demographic variables, lifestyle characteristics and chronic conditions with DS among the older adults in rural setting of Nepal. Our results showed that being female, Buddhist, *Dalits* and *Madhesi* ethnicity, unemployed, low family income, the presence of multiple morbidity and smoking are significant risk factors for DS among older adults in Nepal.

Nearly 55.8% of the older adults were found to be suffering from depressive symptoms. This is lower than reported from other studies in Nepal [7, 18]. A possible explanation for the heterogeneity of prevalence in Nepal could be that different study settings were used, or samples were drawn from diverse ethnicities and castes from the southern plain region were included. The prevalence was also higher than that of a community-based study conducted in rural setting of India (37.8%) and Bangladesh (45%) [4, 20]. In support of our findings, research from low and middle income countries noted a higher proportion of depression among the older population than that of high income countries such as USA, Canada and Australia [21–24].

Our study found that, social determinants (such as gender, religion, ethnicity, income and occupation) were associated with DS and females were more likely to have DS. This is consistent with findings reported by Chalise and Rai from Nepal where authors reported females were more depressed than their male counterparts [25]. This is also supported by the findings from a cohort study that showed a clear link between depressive mood and female gender [26].A possible explanation for this could be that females in a patriarchal society in Nepal may have low self-esteem, low social status and empowerment, feeling helplessness, low health literacy, a longer life expectancy and limited access to health services compared to the males. This is supported by the low position of Nepal in the gender inequality index 0.476 [115th position] in the world, which depicts the disparity of health across genders [27]. In the present study, the subjects who ascribed to Buddhism religion was also found to be more depressed, which could be because of the poor socio-economic status of this minority group in the study setting. However, we are cautious in interpreting this result because of the small number of participants (*n* = 19 to *n* = 794) in this group. Moreover, we could not find any other studies confirming this association.

In contrast to another study from Nepal [7], we found smoking was associated with DS in our study population. Our finding is in line with the findings to that from China and Japan [10, 28]. The underlying mechanism linking smoking and DS are complex: Nicotine has antidepressant properties that release dopamine in the mesolimbic reward pathway, which in turn elevate the mood and alleviate stress [29]. However, evidence suggests that smoking plays an important role in changing neurophysiology that increases a smoker’s risk of DS [30]. It is also evidenced that smoking is the vascular risk factor for vascular depression [31]. Additionally, one author mentioned that association between smoking and mental disorders are a result of shared environmental and genetic factors [30].

Literacy, an important socioeconomic factor, was not found to be significant with the occurrence of DS. In contrast, the studies from various settings showed illiteracy as a strong predictor of DS [5, 7, 32]. The underlying reason for this discrepancy is not clearly understood. Unemployment and insufficient family income were both associated with DS. The possible explanation could be that older adults engaged in low income occupation might have led to financial constraints in their life, eventually leading to depression. Adding to this, 2019 Human Development Index has revealed that 34% of the Nepalese population is multi-dimensionally poor and 23% is vulnerable to multidimensional poverty which shows that the country has a deep chasm to fill when it comes to addressing health equity [27]. Meanwhile, in the Nepalese context, most of the older adults depends on family members/caregivers for their daily needs, where families tend to provide better care if the older adults have some economic resources. This situation may turn to increased conflict at the family level, which in turn puts older populations at risk of mistreatment, which may increase the risk of depression [16, 33]. However, in this current study we haven’t accessed the association between elder mistreatment and depression, and we suggest the need of future studies to check this hypothesis.

In the Nepalese context, caste/ethnicity has been a central feature to describe the level of poverty, poor health literacy and health status. In this light, our study demonstrated that the risk of DS was two times higher in *Dalits* compared to those of higher caste. Emerging evidence shows that *Dalits* experience a wide range of social and economic discrimination at various levels (poor living conditions, poor nutrition, low literacy, poor empowerment, poor access to health services and stigmatization) and this results in a severe form of health inequalities [15, 33, 34]. Stigmatization is pervasive and worsens psychological stress for those in the *Dalit* communities [35].

The prevalence of depression among the older population suffering from chronic multi-morbidities was significantly higher (71.55%) than those with one or fewer conditions. This is consistent with findings from other studies [36, 37]. This is the first study from Nepal to report an association between multi-morbidity and DS among Nepalese older adults. This finding is consistent with a meta-analysis that showed chronic illness to be a major risk for depression among older adults [38]. Evidence has shown that people among whom depression co-existed with multi-morbidity may have more functional impairment, poorer quality of life and increased mortality [39]. Depression impairs independence in the older population and, over time, worsens functional outcomes among the multi-morbid group. The decline in functional status may make them more dependent and vulnerable to mistreatment.

Our findings underscore the need for programs to detect, prevent and manage DS in these groups. More specifically, programs need to adopt a population-based approach that includes screening and diagnosis of DS among older adults, mental health literacy of patients and use of evidence-based practices to manage the DS in the community setting particularly focusing on women and marginalized ethnic minorities. In addition, the Government of Nepal is in the process of scaling up the “Package of Essential Non-Communicable disease (PEN)” throughout Nepal. This is a great opportunity to consider the establishment of health and wellness centers under this program to encourage participation of older adults in yoga, relaxation techniques and meditation, which may be useful for good psychological health. Furthermore, peripheral health care professionals, community health workers, psychologists and psychiatrists need to work together to reach and treat older adults with depression in rural Nepal.

Strengths of our study include its large sample size and high response rate (more than 95%). Another strength of this study includes the strong methodology and use of locally trained enumerators for data collection. However, our study is subjected to certain limitations too. All the associations in this study were cross sectional and cannot evaluate causality. There is a need for longitudinal studies to establish this casual pathway and evaluation of interventions that could address the impact multi-morbidity. As this study only involves the participants from two districts of Nepal, findings cannot be generalized to other setting of Nepal. Another limitation was the use of self-reported data, where social-desirability bias may have occurred.

## Conclusions

A large proportion of the Nepalese older populations were found to have DS, and these were associated with socio-demographic characteristics, lifestyle factors and the presence of multi-morbid conditions. Our findings emphasize the need for programs to detect, prevent and manage depression among the older adults in the community setting. We also suggest the need of targeted screening for DS in the clinical setting focusing on high risks groups such as older adults with multiple morbidities or any chronic conditions, stressful environments or social isolation. This may help health professionals to intervene early in order to avert worsening of the condition.

## Data Availability

The datasets used and/or analyzed during the current study are available from the corresponding author on reasonable request.

## Abbreviations

DS: Depressive symptoms
COPD: Chronic Obstructive Pulmonary Disease
CVD: Cardiovascular Disease
CI: Confidence Interval
aOR: Adjusted Odds Ratio
OR: Odds Ratio
WHO: World Health Organization
RMs: Rural Municipalities
